# Estimating the Impact of Aging on Visual Function Using Useful Field of View (UFOV) with a Focus on the Population of Gangwon-do in Korea

**DOI:** 10.3390/brainsci15010029

**Published:** 2024-12-29

**Authors:** Sang-Bin Na, Seong-Youl Choi, Da-Bin Jeon, Soo-Jin Moon, Jin-Keun Kim

**Affiliations:** Department of Occupational Therapy, Kangwon National University, Samcheok-si 25949, Gangwon-do, Republic of Korea; nsb0526@naver.com (S.-B.N.); davinci0327@naver.com (D.-B.J.); dpdlal0719@naver.com (S.-J.M.); wlsrms1112@naver.com (J.-K.K.)

**Keywords:** aging, driving, elderly people, later-middle-aged, visual function

## Abstract

Background/Objectives: There is a need in Korea for research estimating the impact of aging using the Useful Field Of View (UFOV) test, which can evaluate visual function for elderly drivers. Methods: This observational study involved young people in their twenties and thirties, later-middle-aged people in their fifties or older, and elderly people 65 or older recruited from the Gangwon-do region. UFOV testing was conducted on the participants where the participants completed a questionnaire about general and driving-related characteristics. A one-way analysis of variance (ANOVA) was performed to analyze the mean difference by age group, and a Pearson correlation analysis was carried out to evaluate the correlation between age and visual function. In addition, a simple linear regression analysis was conducted to verify UFOV subdomains that can confirm changes according to age increasing. Results: Findings after analyzing UFOV subtest differences by age group revealed significant differences in the visual function index of the young, later-middle-aged, and elderly in all three tests, and the difference between the later-middle-aged and old groups was only found in divided attention. The correlation between age and visual function was significant in all three subtests. And all three subtests were confirmed to be indicators that can verify changes according to increasing age. Conclusions: This study showed that visual function significantly decreases with age. Selective attention was confirmed as a visual function type that changes sensitively according to increasing age.

## 1. Introduction

The main population indicators of the Korean Statistical Information Service (KOSIS) in 2023 indicated that Korea’s old-age population was increasing—the total population was decreasing after 2020, while the elderly population aged 65 or older in 2023 increased by 2.7% compared to 2020 [1]. According to the Korea Road Traffic Authority’s Traffic Accident Analysis System (TAAS) in 2022, the total number of driver’s license holders in 2020 increased by approximately 2.8% from 33,190,565 to 34,133,763 in 2022, and, among them, the percentage of driver’s license holders aged 65 or older increased by 12.8% compared to 2020 [2]. Surprisingly, the number of accidents caused by elderly drivers aged 65 to 70 years old increased by 13.5% in 2022 compared to 2020, and the number of accidents caused by elderly drivers aged 71 years or older increased by 9.06% [3]. This shows that the number of traffic accidents increases as age increases. According to Sim et al. (2009), the percentage of traffic accidents increases with age, and those in their seventies had a higher rate of serious accidents than those in their sixties. Delays in perceptual reaction time, declines in sensory and physical functions, and declines in cognitive and reaction abilities that appear with age are considered to be essential factors [4]. Therefore, the crucial information in the study of Sim et al. (2009) is that elderly drivers have more serious accidents than younger drivers. In other words, they do not adequately compensate for their decreased functionality [5]. This leads to restricted mobility due to the inability to participate in relatively difficult driving tasks caused by old age [5]. This fact supports the need to provide training for the safety of elderly drivers.

The elderly’s right to mobility is a necessary foundation for their quality of life. For elderly people, driving leads to an independent life, expands the scope of their daily activities, and promotes active participation in social activities, which all help improve the quality of life [6]. Driving expands the range of movement and activities of older people and, hence, plays a crucial role in individual social abilities and autonomy [7]. In addition, it enriches daily activities, such as leisure and religious life, and helps the individual lead a comfortable life through various interactive activities [8]. Therefore, driving can be seen as an essential task that supports active life and leisure in retirement [5].

As the elderly population is increasing, the participation of older people in driving also increases. Actually, in Korea in 2022, driver’s license holders aged 65 or older increased by 12.8% compared to 2020 [1]. Additionally, in 2022, the accident rate and road deaths per 10,000 elderly people in Korea were 4.3 and 1.4, respectively, and the risk of traffic accidents among elderly people in Korea was higher than the Organization for Economic Co-operation and Development (OECD) average. These older drivers had a higher crash involvement rate compared with the younger drivers; therefore, a management solution is needed for the elderly population [9].

However, due to physiological aging, human physical functions such as flexibility, coordination, and movement speed, and cognitive functions, such as short-term memory, concentration, and judgment, gradually decline and may also change due to disease or injury [10]. Elderly drivers’ various social participation activities may be limited due to aging. Among them, driving is a personal means of transportation for the elderly to move around the community, and restrictions on driving participation in old age are a risk factor that hinders the elderly’s overall work participation, including integration into the community [11]. Even Korean elderly workers cannot avoid the ‘rush hour’ because they work at the same time as younger people. Therefore, South Korea also needs strategies to manage and monitor elderly driving function, to prevent the risk of accidents from the elderly driver participating in the community.

In the United States, the number of traffic accidents involving elderly drivers is expected to increase by 178% between 2005 and 2030 [12]. Elderly drivers drive shorter distances than non-elderly drivers but have a higher percentage of vehicle crashes [9]. As cognitive abilities decline, more time is spent processing and reacting to environmental changes [13]. In Korea, the accident rate of elderly drivers has also increased by 25% over the past five years [3]. In addition, it has been reported that, in Korea, the later-middle-aged already begin to feel burdened by driving, and many life restrictions occur due to driving limitations in the elderly [5]. Therefore, the continuous management of driving ability is necessary from later middle age.

Driving is a complex task that requires visual perception, cognition, and motor skills [14]. In particular, while the vehicle moves correctly while driving, the surrounding environment also changes rapidly, which must be checked and reacted to [15]. The driver also faces the challenge of simultaneously watching ahead and operating the vehicle while operating the vehicle’s console box, navigation, air conditioner, radio, etc. [16]. Aging naturally reduces visual and cognitive abilities, which makes it difficult to perform these complex driving tasks [10]. As a result, visual attention on the surrounding environment by looking front and simultaneously looking ahead is an important factor that needs sustained checking of the change caused by aging.

Many assessment tools can evaluate one’s driving ability: the PADA-AD test, a driver’s handover ability evaluation tool; [17]. MMSE, a cognitive function evaluation tool; ADS, a memory evaluation tool; WAIS-III Digit Span, a working memory evaluation tool; the Rey Complex Figure Test, which involves remembering an example, drawing from memory without an example, and then drawing the remembered example again 15 min later; the Trail Making Test (TMT), a screening test for stroke drivers that involves looking at 20 photos of traffic signs and matching signs to the presented traffic situations, consisting of parts A and B, which are widely known for assessing driving suitability; and the Useful Field Of View test (UFOV), an evaluation tool for selective, divided, and simultaneous attention. Among them, UFOV was confirmed to be the most relevant evaluation tool for driving suitability [18].

In a study examining the effects of age, gender, alertness, education level, and hearing sensitivity on visual and verbal working memory and processing speed, visual and verbal working memory and processing speed appear to decrease as age increases [17]. Additionally, in a study comparing the visual–motor processing speed and reaction time of healthy adults according to age and gender, elderly people showed slower visual–motor processing speed and reaction time [19]. As a result of conducting a UFOV test for the elderly in the study, it was suggested that UFOV standardized data could be provided and that these data could be a vital indicator of driving performance while verifying that UFOV is a test that is less affected by external factors, such as education, visual function, self-rated health, and health and mental status [20].

Hence, screening changes in driving-related functions such as visual attention, executive function, general condition, memory, spatial perception, visual closure, contrast sensitivity, visual procession speed, and visual acuity ability, which changes sensitively with age, through a certified test such as UFOV effectively predicts the risk of health deterioration in the elderly population and participation in high-level activities such as driving. In particular, UFOV subtests 1, 2, and 3 symbolically evaluate the visual processing speed, divided attention, and selective attention, respectively [18]. These functions are also proposed as important indicators to measure visual function related to driving performance [20]. From this perspective, UFOV tests are known to be highly related to driving. In particular, UFOV tests provide more objective results by analyzing the driver’s response to changes in stimulus display time through a computer rather than simply measuring the driver’s response speed [18]. Due to these advantages, the UFOV test is standardized and utilized in various countries [21]. Accordingly, there is a need to continue efforts in Korea to confirm the effect of aging for the visual function test related to driving for the elderly, using UFOV to improve the usability of the test.

Therefore, this study aims to compare the visual function related to driving of young, later-middle-aged, and elderly people through the UFOV test, and identify elements of visual function that respond more sensitively with age.

## 2. Materials and Methods

### 2.1. Study Design and Process

This observational study conducted experiments from 28 November 2022 to 31 January 2023. The test was conducted at the driving rehabilitation laboratory at Gangwon National University in Gangwon-do. The selected participants completed a questionnaire containing general and driving-related characteristics and then underwent a UFOV test. The results were derived by measuring one time with two consecutive tests for each individual to collect objective results from the participants during the test.

### 2.2. Participants

This research included 78 young people in their twenties and thirties, 22 later-middle-aged people in their fifties or older, and 38 elderly people in their 65s or older living in Gangwon-do. Considering the mobility of the elderly, additional recruitment was conducted through snowball sampling, focusing on participants recruited after the announcement of the research conduct in the research environment. After participating in the preliminary survey, two participants withdrew their participation in the experiment due to personal reasons. Hence, a total of 138 participants completed the final experiment. The size of the sample was calculated through the G-power program. Based on 3 groups, large effect, and 95% confidence level, 102 appropriate samples were identified for the mean difference test of the one-way distribution analysis. Therefore, samples above this value were recruited. Nevertheless, the number of later-middle-aged and elderly participants recruited was smaller than that of young participants. In order to randomly sample those who need help related to driving, the participants were recruited through public announcement, but, unfortunately, the support for later-middle-aged and elderly was small. Because of the fact that the Gangwon-do region has the characteristics of being mountainous and having a narrower local road, the data from this region are remarkable. The selection criteria for study’s participants are as follows:

- Those who voluntarily participated in the study;
- Those who do not have any visual problems and ocular disorders that may affect the results of the test;
- Those who understand the investigator’s verbal instructions and can perform the UFOV test;
- Healthy adults without specific diseases including neurological or musculoskeletal disorders;
- Those who have no prior experience conducting UFOV inspections.

Information related to the subject was collected through a questionnaire. Considering the age of the subject, the subject filled out the questionnaire with the researcher’s question. The questions included in the questionnaire were the subject’s general characteristics, disease-related information selection, vision problems, etc. The participants entered their names and birth dates in the UFOV subject information and attempted sample questions for each subtest to confirm that they fully understood the study before proceeding with the test. This study complies with the Declaration of Helsinki and was performed with the approval of the Kangwon National University Bioethics Review Committee for the entire research process (approval number: KWNUIRB-2022-11-006-001). Before participating, the participants were oriented of the study’s purpose, entire process, and treatment and compensation in case they suffered harm as a result of participation. In accordance with the research ethics of the Institutional Review Board (IRB), written consent was obtained from the participants to ensure that they acknowledge the study’s purpose, method of data use, information destruction upon completion of the study, collection of personal information, and confidentiality.

### 2.3. Measures

#### Useful Field of View Test (UFOV)

UFOV was developed by Karlene Ball in 1988 to address the need for visual perception and cognitive evaluation in driving performance. UFOV (Visual Awareness Research Group Inc., Sarasota, FL, USA) evaluates visual perception and visual function by monitoring (touch-screen version, 17-inch, refresh rate 60 Hz) them through a software program (version 6.1.4). Its subdomains include visual processing speed, selective attention, and divided attention [22].

The test comprises three stages, and the level of difficulty increases as each stage progresses (e.g., screen transition speed and peripheral vision obstacles increase). The first subtest evaluates visual processing speed by recognizing the central visual field as the target, the second subtest examines processing and divided attention in central and peripheral vision, and the third subtest has the same method as the second subtest but assesses selective attention to accurately find desired visual information in situations where there are obstacles around.

In UFOV test, the object was presented to the short brief display time (16.67–500) via the double-step-case method. According to the manual, the test was performed sitting on a chair so that the distance between the subject’s viewpoint and the monitor was 21 inches. The subject was instructed to look at the screen and fix the head, and, if there was movement, the posture was corrected using the break time of the test. In the first UFOV subtest, a 2 cm × 1.5 cm-sized truck or sedan-type vehicle appears in a 3 cm box in the center (fixation box), and participants identify what type of vehicle it is. Therefore, it can measure the visual processing speed. The second subtest involves the identification of a central target and a vehicle is displayed at a random location in eight directions (0°, 45°, 90°, 135°, 180°, 225°, 270°, and 315°). Participants identified the type of vehicle of the central target and which of the eight directions the vehicles was located in. Therefore, it can assess the divided attention. The third subtest is the same as the second subtest, but 47 triangles of the same size and brightness as the vehicles are added. Therefore, it can test selective attention. The evaluation results are stored as milliseconds (ms), which is the reaction time to the program.

In a previous study that verified reliability and validity in Korea, test–retest reliability was high at 0.83 for subtest 1, 0.90 for subtest 2, and 0.93 for subtest 3, and inter-investigator reliability was very high at 0.92, 0.96, and 0.99 for subtests 1, 2, and 3, respectively. The concurrent validity with TMT A&B and MVPT(VC) was high at 0.77 and 0.85, and the discriminant validity of visual attention reduction in stroke patients was significant for subtests 1, 2, and 3 [23].

### 2.4. Statistical Analysis

The study’s collected data were analyzed using IBM SPSS Statistics ver. 26.0. The chi-square analysis was performed using descriptive statistics to analyze the general characteristics of young people, later-middle-aged people, and elderly people, and a factorial ANOVA was carried out to determine the difference in means by age group and driving license state. Pearson correlation coefficient was performed to analyze the correlation between age and visual function. Because of the small sample size in each group, we cannot construct multi-variable regression analysis. Therefore, a simple linear regression analysis was performed to confirm the change in visual function according to increasing age and driving experience. And, to compensate for the relatively small sample of later-middle-age and elderly age, and large deviation in UFOV scores, the scores of the three subtests were converted to logs and additional regression analysis was performed. Mean and median analyses were conducted to analyze data by age, and the significance level α for all analyses was 0.05.

## 3. Results

### 3.1. The Characteristics of Participants

Table 1 details the general characteristics of the study’s participants. Among the young people, 48.7% were male and 51.3% were female, and the average age was 22.87 years. Among the later-middle-aged, 68.2% were female and 31.8% were male, and the average age was 59.45 years. Among the elderly people, 57.9% were female and 42.1% were male, and the average age was 73.74 years. We found that 59.0%, 77.3%, and 68.4% of the young, later-middle-aged, and elderly, respectively, obtained a driver’s license, and their driving experience was 3.20 ± 1.80 years, 19.65 ± 7.20 years, and 27.73 ± 7.70 years, respectively. In the result of the chi-square analysis of the balance of gender and driving license by age group, no significant difference was identified (Table 1).

### 3.2. Comparison of Averages by UFOV Subdomains According to Age Group and Driving License State

**Figure 1:**
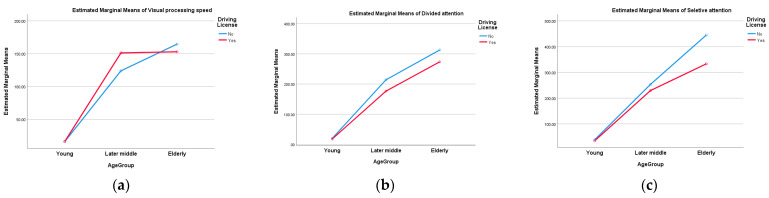
This figure is a graph explaining the factorial ANOVA of the UFOV subtest by increasing age and driving license states: (**a**) estimated marginal means of visual processing speed; (**b**) estimated marginal means of divided attention; and (**c**) estimated marginal means of selective attention.

After comparing the average differences by subdomain according to age group, a statistically significant difference was found in all three subdomains of visual processing speed, divided attention, and selective attention. In the Scheffe test, young people had relatively lower averages than later-middle-aged and elderly people in all three subdomains of visual processing speed, divided attention, and selective attention, and later-middle-aged people had relatively lower averages than elderly people in subdomain 3 (*p* < 0.001) (Figure 1 and Table 2).

### 3.3. Correlation Between Age and Visual Function

A significant correlation between age and the three subdomains was also identified. Additionally, the correlation between subdomains was significant between visual processing speed and divided attention, divided attention and selective attention, and visual processing speed and selective attention (Table 3).

### 3.4. UFOV Subdomain Analysis to Verify Changes with Age

**Figure 2:**
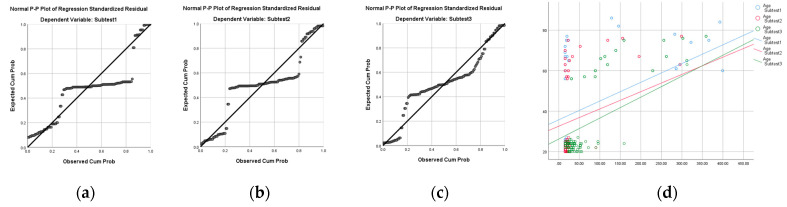
This figure is a graph explaining the simple linear uni-variable regression of the UFOV subtest by increasing age: (**a**) normal P–P plot of regression standardized residual with subtest 1; (**b**) normal P–P plot of regression standardized residual with subtest 2; (**c**) normal P–P plot of regression standardized residual with subtest 3; and (**d**) scatter plot between UFOV subtests and age.

Table 4 shows the results of the simple linear regression analysis to identify the subtest that can distinguish the difference in visual function by increasing age among the three subtests of UFOV. Visual processing speed, divided attention, and selective attention were identified as variables to predict the difference by increasing age (*p* < 0.001). The R^2^ of the uni-variable in visual processing speed with increasing age was 0.28, divided attention was 0.46, selective attention was 0.67 (Figure 2 and Table 4).

**Figure 3:**
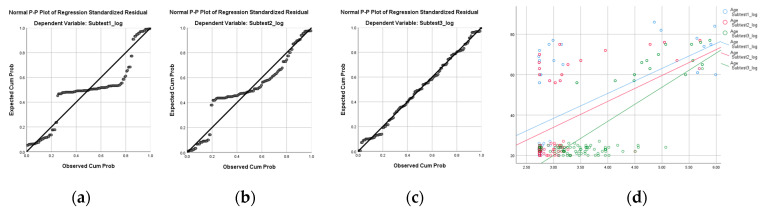
This figure is a graph explaining the simple linear uni-variable regression of the UFOV subtest by increasing age: (**a**) normal P–P plot of regression standardized residual with subtest 1 log data; (**b**) normal P–P plot of regression standardized residual with subtest 2 log data; (**c**) normal P–P plot of regression standardized residual with subtest 3 log data; and (**d**) scatter plot between UFOV subtests’ log data and age.

And Table 5 shows the results of the simple linear regression analysis to the three subtests’ log data of UFOV by increasing age. Visual processing speed, divided attention, and selective attention were identified as variables with which to predict the difference by increasing age (*p* < 0.001). The R^2^ of the uni-variable in the log data of the visual processing speed with increasing age was 0.37, divided attention was 0.55, selective attention was 0.77 (Figure 3 and Table 5).

### 3.5. UFOV Subdomain Analysis to Verify Changes with Driving Experience

**Figure 4:**
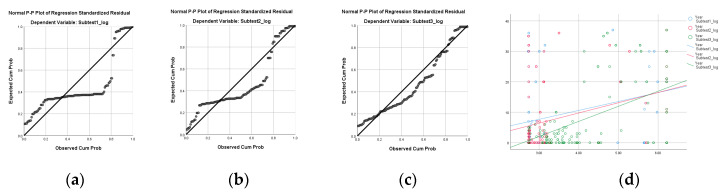
This figure is a graph explaining the simple linear uni-variable regression of the UFOV subtest by increasing age: (**a**) normal P–P plot of regression standardized residual with subtest 1; (**b**) normal P–P plot of regression standardized residual with subtest 2; (**c**) normal P–P plot of regression standardized residual with subtest 3; and (**d**) scatter plot between UFOV subtests and driving experience.

Table 6 shows the results of the simple linear regression analysis to identify the subtest that can distinguish the difference in visual function by increasing driving experience among the three subtests of UFOV. Visual processing speed, divided attention, and selective attention were identified as variables to predict the difference by increasing driving experience (*p* < 0.001). The R^2^ of the uni-variable in the visual processing speed with increasing age was 0.28, divided attention was 0.15, selective attention was 0.26 (Figure 4 and Table 6).

### 3.6. The Data for UFOV Subtests by Age

Table 6 presents the data of UFOV test according to descriptive statistics. The median and average were lowest for young people, followed by later-middle-aged people and elderly people (Table 7).

## 4. Discussion

This study aimed to compare visual perception and analyze UFOV data for healthy young people, later-middle-aged people, and the elderly in Korea, and analyze the correlation and regression between visual function according to age. The results suggested that, as age increases, visual processing speed, selective attention, and divided attention decrease. In particular, selective attention is a crucial aspect in confirming the decline in visual function after middle age.

Through this study, it was confirmed that there were differences in three visual attention spans between age groups. Visual processing speed, divided attention, and selective attention were significantly slower in later-middle-aged and elderly populations compared to younger populations. However, between the later-middle-aged and elderly population, differences were identified only in selective and separative attention, and no differences were identified in visual processing speed. This suggests that selective and separative attention may be more sensitive to changes with aging than to visual processing speed. In a previous study that reported changes in attention according to age increase from 20 to 80, it was confirmed that visual attention gradually decreased with aging [24]. This study reported a remarkable decrease in selective attention, which was confirmed as a problem of reducing the response speed and suppressing impulsiveness. Since UFOV’s subtest 1 is tested in a way that responds to stimuli displayed at one point [18], the impulsiveness of the elderly in actual reactivity can lead to a shortening of time. In conclusion, it can be estimated that UFOV subtest 1 measures the response speed without distinguishing the impulsive suppression variables of the elderly. This is interpreted as a feature of the test that does not require the accuracy of the classification of stimuli. The actual UFOV is focused on the peripheral field of view rather than the accuracy of the central field of view. In addition, the test emphasizes that one subtest should not be interpreted as representing only one ability in a dichotomous way [25]. Being aware of the presence of objects during actual driving can be more important than distinguishing what they are. We should not forget that UFOV inspection was developed around the ability to be highly related to driving. In the factorial ANOVA analysis of this study, it was confirmed that the elderly were less affected by the license status in the case of visual processing speed. On the other hand, it was confirmed that selective attention was more affected by the license status in the elderly population.

The study confirmed that, as age increases from youth to later middle age and old age, the UFOV subdomain score increases and visual function continues to decrease. Similarly, Marks et al. (2015), who conducted a UFOV test on 52 healthy adults (19 to 69 years old), claimed that the UFOV score was lower when the age was younger [22]. Additionally, in a study by Edwards et al. (2006), which conducted a UFOV test on 2759 people aged 65 to 94, the scores of participants aged 65 to 75 were lower than those of those aged 75 or older [20]. Therefore, the results of this study on the correlation between age and visual function were similar to those of previous studies.

The results of Alberti et al. (2014) [26], which confirmed a significant correlation between visual function in the three subdomains of the UFOV test and age increase, are also consistent with the current study’s findings. These studies demonstrate that older adults perform worse in these UFOV subtests due to age-related declines in visual search speed, attention, and the ability to filter out distractors. Additionally, the tendency for older adults to make shorter forward eye movements and fixate closer to target words in reading tasks could further explain their slower performance. Including this would provide a broader context for the performance patterns seen in the UFOV test. On the other hand, the non-significant differences between age groups in visual processing speed of the UFOV could be that peripheral vision is relatively stable through aging [27]. However, there is a certain controversy in this regard. Another potential explanation might be the older participants not maintaining a fixed gaze, instead glancing at the targets to solve the visual processing speed test of the UFOV and, therefore, obtaining similar results compared to younger participants, as reported in a previous study [28]. Considering that divided and selective attention is required in the other two subtests of the UFOV, older participants obtain significantly worse results than younger ones [29]. And, in previous studies, divided attention by age is a factor that can distinguish between young people and later-middle-aged and elderly adults, and the decrease in reaction speed was significant as age increased [30]. In other words, previous studies found results which demonstrated that visual processing speed, selective concentration, and divided attention deteriorate with age. Moreover, the study confirmed that selective attention change is an essential part of increasing age, and visual processing speed, divided attention, and selective attention change were related to age increasing.

The study found the lower significant causality with age of visual processing speed of the UFOV. These findings suggest that peripheral vision remains relatively stable with age. While there is some controversy in this area, the stability in peripheral vision might explain why older adults performed similarly to younger participants on this subtest. This would provide a deeper understanding of why visual processing speed might not deteriorate as significantly in some aspects [31]. It suggests that older participants may not keep their gaze fixed during the visual processing speed test of the UFOV. This could explain why there are similar results between older and younger groups. Older individuals might glance at targets instead of focusing on a central point, which could impact their performance on this subtest. In other words, this means that the central information perception of older drivers can be relatively preserved despite the age increase. Although the results of the simple regression analysis have limitations in not considering the effects of other variables, in this study, three visual attentions are affected as age increases, and divided and selective attention are greatly affected compared to visual processing speed. On the other hand, it was found that driving experience has the most influence on visual processing speed. Older people who are bound to have relatively more driving experience due to their age may also be affected by these variables. However, the accuracy of the information judgment of elderly drivers may be degraded. Therefore, it is necessary to investigate the effects of more variables affecting the elderly through more research.

In a study that examined the reaction speed of 30 young and 30 elderly people, the reaction speed of the young group was found to be faster than that of the elderly [32]. Additionally, in a work of research comparing the selective attention of 20 young and 20 elderly participants, the elderly participants had slower reaction speeds on average, but no significant differences were found [33,34]. These existing research results, together with the results of this study, support that reaction speed decreases with general aging. However, this study differs from existing studies by confirming the correlation between increasing age and slowing reaction speed and specifying that selective attention is a major change factor in aging.

During the normal aging process, neurological damage and visual perception degeneration limit active participation or mobility in daily activities. Although this change in visual perception ability is a factor that influences the loss of many functions, it is not managed and is often accepted as a natural change due to aging; thus, preventive management is important [35]. Accordingly, in a society where aging is accelerating, the continuous examination and management of visual function changes in the elderly is an essential health policy. Considering these aspects, the sensitive predictability of changes in visual function after later middle age and the data on visual function in the elderly population identified in this study can be useful in terms of preventive health.

Changes in visual function are highly reliable in predicting changes due to aging, and many normative data analyses and standardization studies of UFOV examinations have already been reported in various countries [20]. Furthermore, this assessment is a highly correlated tool for assessing driving suitability in the elderly population [18]. By comparing the performance of UFOV between younger and older people, it was confirmed that time–space attention decreased as age increased in Korea [32]. But the studies that presented the data and reported the causality of age increase and visual function are lacking. The age-specific visual function scores and the causality presented in this study can be used as data for future visual function tests for Korean elderly. But we hope that this study will serve as a starting point for continued related research because the size of populations in the study is small.

Previously, studies that provided UFOV standard data according to the education level of Caucasian and African American in the United States have been reported [20]. The mean score over 85 years of age with a 12-year education level of the UFOV selective attention presented in previous studies was similar to that of the elderly over 65 years of age in Korea in the study. In addition, the median of the study was found to be relatively high compared to a study that provided UFOV standard data for elderly drivers over 70 years of age in a previous study that reported Swedish standards [36]. Compared to Sweden and the United States, which previously reported long-term data of more than 2000 people, this study is judged to have a difference due to the recruitment of participants in a limited area for a short period of time. Moreover, the fact that 80% of the participants aged 65 and over are in their seventies and eighties are considered to be affected. In addition, it should be noted that the existing studies have a larger proportion of males in the sample, while this study has a small proportion of males, and most of them live in mining areas and have poor educational standards. Although the results of a simple regression analysis have limitations in not considering the effects of other variables, in this study, three visual attentions are affected as age increases, and divided and selective attention are greatly affected compared to visual response speed.

This study presented UFOV data for adults in Korea, the target group of previous studies. Although the research data were collected only in Gangwon-do, it has the largest number of mountains and narrower local roads in Korea, making driving difficult. Therefore, the data of elderly drivers in this study are noteworthy. And, among the subdomains of UFOV, visual function changed sensitively with age, especially in subdomain 3 (selective attention), which is considered an important indicator for the early screening of visual function deterioration in elderly people.

The limitations of this study are as follows: First, due to limitations in research facilities, snowball sampling was conducted only on young people, later-middle-aged people, and the elderly in the Gangwon-do region, making it difficult to generalize the research results to the entire Korean population. However, considering the characteristics of the Gangwon-do region, the research data are remarkable. Second, there is a limitation in recruiting people by age group equally during the promotional recruitment and snowball sampling process. Third, this study was conducted on healthy adults, so data on populations with specific diseases were not obtained. Finally, the number of samples was small and long-term follow-up was not achieved because the year of Covid was included. Despite these limitations, this study is significant in that it is the first study to provide Korean UFOV data. Therefore, we hope that various studies in which variables are controlled for large samples with evenly distributed ages will continue in the future, considering the above limitations. In addition, a longitudinal study of the elderly population in Korea could also be conducted.

## 5. Conclusions

This study aimed to compare the visual function of young, later-middle-aged, and elderly people through the UFOV test, and present the validity and data to compare attention problems after the UFOV test. The main findings identified in this study are as follows:

1. The visual function that becomes most sensitive with age is selective attention;
2. Changes in visual processing speed, divided attention, and selective attention according to increasing age can be verified through the UFOV test;
3. Compared to young people, visual processing speed, selective attention, and divided attention all tended to decrease as age increased, especially for later-middle-aged and elderly people.

## Figures and Tables

**Table 1:** Characteristics of participants.

Characteristics	Values			x^2^/F	*p*
	Young Age	Later-Middle-Age	Elderly Age		
Sex, n (%)				2.89	0.24
Male	38 (48.7)	15 (68.2)	22 (57.9)		
Female	40 (51.3)	7 (31.8)	16 (42.1)		
Age (years), mean (SD)	22.87 (1.8)	59.45 (2.26)	73.74 (6.2)	2846.71	<0.001
Driver license, n (%)				2.86	0.24
No	32 (41.0)	5 (22.7)	12 (31.6)		
Yes	46 (59.0)	17 (77.3)	26 (68.4)		
Driving experience (years), mean (SD)	3.20 (1.8)	19.65 (7.2)	27.73 (7.7)	55.27	<0.001

**Table 2:** Comparison of visual function among age groups and driving license state.

Visual Function		Mean ± SD (ms ^1^)	Driving License		F		Scheffe
Visual processing speed			No	Yes			
	Young people ^a^	16.19 ± 1.67	16.09 ± 1.47	16.26 ± 1.80	Age	19.76 ***	a < b,c
	Later-middle-aged ^b^	144.99 ± 181.59	123.90 ± 147.89	151.19 ± 193.98	License	0.04	
	Elderly people ^c^	156.63 ± 178.07	164.62 ± 185.73	152.95 ± 174.46	License × Age	0.14	
Divided attention			**No**	**Yes**			
	Young people ^a^	18.85 ± 8.86	20.09 ± 13.27	18.00 ± 3.36	Age	43.15 ***	a < b,c b < c
	Later-middle-aged ^b^	185.13 ± 227.73	214.04 ± 261.04	176.62 ± 225.15	License	0.67	
	Elderly people ^c^	286.02 ± 219.50	312.88 ± 215.48	273.62 ± 220.10	License × Age	0.23	
Selective attention			**No**	**Yes**			
	Young people ^a^	35.88 ± 22.50	38.31 ± 19.80	34.19 ± 24.26	**Age**	121.11 ***	a < b,c b < c
	Later-middle-aged ^b^	235.03 ± 197.08	253.52 ± 225.00	229.59 ± 195.41	**License**	3.67	
	Elderly people ^c^	368.61 ± 155.97	445.17 ± 82.21	333.27 ± 167.16	**License × Age**	2.72	

^a–c^ the result of Scheffe analysis. ^1^ ms; millisecond. *** *p* < 0.001.

**Table 3:** Correlation between visual function and age.

	Age (Years)	UFOV ^1^		
		Visual Processing Speed	Divided Attention	Selective Attention
Age (years)	1	-	-	-
Visual processing speed	0.53 **	1	-	-
Divided attention	0.68 **	0.86 **	1	-
Selective attention	0.82 **	0.78 **	0.92 **	1

^1^ UFOV; Useful Field Of View Test. ** *p* < 0.01.

**Table 4:** UFOV subtest predictors by increasing age—simple linear uni-variable analysis.

Dependent Variables	Estimate	95% Confidence Interval		*p*	R^2^
		Lower Limit	Upper Limit		
Visual processing speed	3.04	2.21	3.86	<0.001	0.28
Divided attention	5.41	4.42	6.40	<0.001	0.46
Selective attention	6.49	5.72	7.26	<0.001	0.67

**Table 5:** UFOV subtest log predictors by increasing age—simple linear uni-variable analysis.

Dependent Variables	Estimate	95% Confidence Interval		*p*	R^2^
		Lower Limit	Upper Limit		
Visual processing speed	0.03	0.02	0.04	<0.001	0.08
Divided attention	0.04	0.04	0.05	<0.001	0.55
Selective attention	0.05	0.04	0.05	<0.001	0.77

**Table 6:** UFOV subtest log predictors by increasing driving experience—simple linear uni-variable analysis.

Dependent Variables	Estimate	95% Confidence Interval		*p*	R^2^
		Lower Limit	Upper Limit		
Visual processing speed	0.03	0.01	0.44	<0.01	0.28
Divided attention	0.04	0.03	0.06	<0.001	0.15
Selective attention	0.05	0.04	0.07	<0.001	0.26

**Table 7:** UFOV subtest score for each age group. Unit: milliseconds.

Useful Field of View Test		Median	Total (Mean ± SD)	Participants with Driver License (Mean ± SD)	Participants Without Driver License (Mean ± SD)
Visual processing speed					
	Young people	15.50	16.19 ± 1.67	16.26 ± 1.80	16.09 ± 1.47
	Later-middle-aged	18.70	144.99 ± 181.59	123.90 ± 147.89	151.19 ± 193.98
	Elderly people	23.90	156.63 ± 178.07	152.95 ± 178.06	164.62 ± 194.80
Divided attention					
	Young people	15.90	18.85 ± 8.86	18.00 ± 3.36	20.08 ± 13.27
	Later-middle-aged	23.05	185.13 ± 227.73	176.62 ± 225.15	214.04 ± 261.05
	Elderly people	298.90	286.02 ± 219.50	273.62 ± 224.63	312.88 ± 226.00
Selective attention					
	Young people	29.95	35.88 ± 22.50	34.19 ± 24.26	38.31 ± 19.80
	Later-middle-aged	118.80	235.03 ± 197.08	229.60 ± 195.41	253.52 ± 225.00
	Elderly people	355.50	368.61 ± 155.97	333.27 ± 170.61	445.17 ± 86.23

## Data Availability

The data are not publicly available because of ethical and legal regulations regarding the protection of personal data.
